# Incidence rates of prediabetes and diabetes associated with sedentary behavior and night shifts among peruvian workers (2014–2021)

**DOI:** 10.1016/j.dialog.2025.100204

**Published:** 2025-01-06

**Authors:** Víctor Juan Vera-Ponce, Fiorella E. Zuzunaga-Montoya, Nataly Mayely Sanchez-Tamay, Juan Carlos Bustamante-Rodríguez, Carmen Inés Gutierrez De Carrillo

**Affiliations:** a Instituto de Investigación de Enfermedades Tropicales, Universidad Nacional Toribio Rodríguez de, Mendoza de Amazonas (UNTRM), Amazonas, Peru; b Facultad de Medicina (FAMED), Universidad Nacional Toribio Rodríguez de Mendoza de, Amazonas (UNTRM), Amazonas, Peru; c Universidad Continental, Lima, Peru

**Keywords:** Occupational medicine, Occupational health, Night shift work, Sedentary behavior, Diabetes mellitus, Prediabetic state (source: MeSH NLM)

## Abstract

**Introduction:**

Type 2 diabetes mellitus (T2DM) and prediabetes represent a global public health concern, with increasing prevalence in developing countries. Occupational factors such as sedentary behavior and night shift work may play a significant role in their development; however, there is limited information on their impact on Latin American populations.

**Objectives:**

To determine the incidence of T2DM and prediabetes and to evaluate the association between prolonged sitting time and night shift work with glycemic changes in Peruvian workers.

**Methods:**

A retrospective cohort study was conducted with 4200 workers evaluated between 2014 and 2021. Incidence rates of T2DM and prediabetes were calculated, and Cox regression models were used to assess the association between prolonged sitting time and night shift work with glycemic changes. The measure of association was the crude and adjusted hazard ratio (aHR), presented with its respective 95 % confidence interval (95 % CI).

**Results:**

The incidence of T2DM was 33.1 per 1000 person-years, and that of prediabetes was 77.11 per 1000 person-years. Sitting time (≥ 4 h/day) was associated with a higher hazard of diabetes (aHR: 2.84, 95 % CI: 1.58–5.12). Night shift work also significantly increased the hazard of diabetes (aHR: 3.24, 95 % CI: 1.97–5.35).

**Conclusion:**

This study reveals a high incidence of T2DM and prediabetes among Peruvian workers, with significant associations between prolonged sitting time and night shift work with glycemic changes. The results underscore the importance of considering these occupational factors in T2DM prevention strategies. Implementing workplace prevention and early detection programs focused on reducing sedentary time and mitigating the effects of night shift work is recommended.

## 1 Introduction

Type 2 diabetes mellitus (T2DM) and prediabetes represent a growing global public health concern. According to the International Diabetes Federation, an estimated 537 million adults will be living with diabetes in 2021, with projections indicating this number will increase to 783 million by 2045 [1]. Prediabetes, a state of elevated risk for developing T2DM, affects an even more significant number of individuals and often goes undiagnosed [2]. In Latin America, the prevalence of diabetes has increased significantly in recent decades, with considerable variations among countries and population subgroups [3].

The work environment plays a crucial role in employees' health, including the risk of developing metabolic diseases such as T2DM and prediabetes. Occupational factors such as job type, working hours, shift work, and sedentary behavior in the workplace have been associated with an increased risk of developing these conditions [4]. A recent meta-analysis found that shift work was associated with a 40 % increase in T2DM risk [5]. At the same time, another study demonstrated that prolonged sedentary time at work positively correlated with higher fasting glucose levels [6].

In Peru, as in many developing countries, the epidemiological transition and changes in lifestyles have led to an increase in the prevalence of non-communicable diseases, including diabetes [7]. However, data on the incidence of T2DM and prediabetes in the Peruvian working population are limited, and studies examining specific occupational risk factors in this context are even scarcer.

Early identification of individuals at risk of developing T2DM or prediabetes is crucial for implementing effective prevention strategies. Work environments offer a unique opportunity for early detection and intervention, given that working-age adults spend a significant portion of their time at work. In particular, prolonged sedentary behavior and night shift work have emerged as potentially modifiable occupational risk factors that warrant special attention.

Thus, this study aims to determine the incidence of T2DM, prediabetes, and glycemic changes in a cohort of Peruvian workers and evaluate the association between prolonged sitting time and night shift work with these changes over time. By focusing on these two specific occupational factors, we seek to provide crucial evidence to inform diabetes prevention and management strategies in the workplace in Peru and, potentially, in similar contexts across Latin America. Understanding how these factors influence diabetes risk can guide the development of more effective and targeted workplace interventions.

## 2 Methods

### 2.1 Study type and design

This study was designed as a retrospective cohort based on a secondary analysis of a database of workers evaluated at an occupational health clinic between 2014 and 2021. The design and presentation of results followed the STROBE (Strengthening the Reporting of Observational Studies in Epidemiology) guidelines to ensure clear and comprehensive reporting of observational research [8].

### 2.2 Population, sample, and eligibility criteria

The initial cohort consisted of workers from various companies who attended periodic occupational evaluations at the mentioned clinic. To be included in the study, workers had to have at least two evaluations separated by a minimum interval of one year. Additionally, they were required to have a complete record of all variables of interest for the research, both at the beginning and end of the cohort.

For the analysis of different events of interest, specific selection criteria were established for each state of glycemic change. In assessing the incidence of T2DM from normoglycemia, only participants with normal glucose levels at the study's onset were included, excluding those with a previous diagnosis of T2DM or prediabetes. Participants with normal glucose levels at baseline were included for the incidence of prediabetes from normoglycemia, excluding those with a previous diagnosis of prediabetes or T2DM. For the incidence of diabetes from prediabetes, only participants who had a diagnosis of prediabetes at the study's onset were included, excluding those with a previous diagnosis of T2DM. For the incidence of normoglycemia from prediabetes, participants with a diagnosis of prediabetes at baseline were included, excluding those with normal glucose levels or a diagnosis of T2DM. In evaluating the overall incidence of T2DM, all participants who had a previous diagnosis of T2DM, either by self-report or laboratory confirmation at the study's onset, were excluded. Finally, for the overall incidence of prediabetes, individuals who, at the study's onset, had already been diagnosed with prediabetes or T2DM, either through self-report or laboratory diagnostic criteria, were excluded.

It is important to note that each worker underwent periodic evaluations over time, following an established scheme that could vary between annual, biennial, or other intervals defined by company policies or labor regulations. This continuous follow-up allowed monitoring of each participant's health status until their last evaluation was recorded in the database.

In cases where a worker was diagnosed with the condition of interest in the study (prediabetes or T2DM), that participant was removed from follow-up for the specific purposes of this research. Although these workers may have been evaluated again in the future as part of their occupational health routine, their data after diagnosis were not included in the subsequent analysis of our study.

### 2.3 Variables and measurement

The main variable of the study was the diagnosis of T2DM and prediabetes, as well as changes in glycemic status over time. The diagnosis of T2DM was established if the fasting glucose level (FG) was equal to or greater than 126 mg/dL (7.0 mmol/L) by the American Diabetes Association (ADA) criteria [9]. The ADA criterion was considered to diagnose prediabetes as an FG level between 100 and 125 mg/dL (5.6 to 6.9 mmol/L).

Since each evaluated worker had a different follow-up time, the follow-up time variable was measured in years, considering each participant's baseline as 0 years.

The events of interest analyzed in this study were the incidence of T2DM, the incidence of Prediabetes, the incidence of T2DM from Normoglycemia, the incidence of Normoglycemia from Prediabetes, and the incidence of Hyperglycemia from Normoglycemia.

Occupational variables included sitting during the workday (up to 4 or more than 4 h) and whether they performed night work (Yes or No).

Additionally, covariates considered in the study included demographic factors, medical history, and clinical measurements. Demographic variables included sex (categorized as Female or Male) and age. Regarding history and habits, family history of diabetes (Yes or No), current smoking status (Yes or No), and alcohol consumption (Yes or No) were considered. Clinical measurements included systolic (SBP) and diastolic (DBP) blood pressure, waist circumference (WC), body mass index (BMI), blood glucose level, cholesterol level, and triglyceride level. Additionally, occupation type (categorized as Office, Manual or Physical, Customer Service or Sales, Health Professional, or Social Services) and time in current job (less than 1 year, 1 to 4 years and 11 months, 5 to 9 years and 11 months, or 10 years or more) were considered.

### 2.4 Procedures

The evaluation process of workers at the occupational clinic was carried out under different contexts: upon entering the company, during periodic evaluations, or at the end of their employment contract. At the beginning of each evaluation, the worker recorded their data in the clinical history, including demographic and work information.

The nursing staff measured SBP and DBP using Omron brand oscillometric devices. Measurements were taken on the worker's right arm seated, ensuring the arm was at chest level. Three measurements were taken with a five-minute interval between each, and the final blood pressure value was determined from the average of the last two measurements.

Next, an anthropometric evaluation included measuring weight, height, and abdominal waist circumference. Weight was measured using a calibrated electronic scale, and height was determined with the worker barefoot on a flat surface using a stadiometer. Waist circumference was measured with a flexible measuring tape placed at the midpoint between the last rib and the upper edge of the iliac crest.

Subsequently, workers were directed to the laboratory, where blood samples were taken by venipuncture after a fast of 8 to 12 h. These samples were analyzed to obtain glucose, triglyceride, and cholesterol levels.

Finally, each worker was evaluated by an occupational physician, who asked a series of questions to learn about the worker's pathological history and that of their immediate family members, as well as lifestyle habits such as smoking, alcohol consumption, time spent sitting, sleep hours, and frequency of daily physical activity. The doctor also carried out a complete physical examination.

Clinic staff entered all collected information into the worker's clinical history and recorded it in an electronic database. A blinding procedure was unnecessary for this registry, as the data were collected as part of the daily routine and not specifically for research purposes.

### 2.5 Statistical analysis

The database was delivered in Excel format, and after a cleaning and validation process, analyses were conducted using R Studio. First, a descriptive analysis of the characteristics of the study subjects at baseline was performed. For categorical variables, absolute and relative frequencies were presented, while means and standard deviations were calculated for continuous variables.

Then, the incidence density per 1000 person-years of follow-up was calculated for each of the events of interest: the incidence of T2DM, the incidence of prediabetes, the incidence of T2DM from normoglycemia, the incidence of T2DM from prediabetes, the incidence of normoglycemia from prediabetes, and incidence of hyperglycemia from normoglycemia. 95 % confidence intervals (95 % CI) were provided for all these estimates. Additionally, survival curves were generated for each of these six states of glycemic change.

For the analysis of occupational variables (sitting time and night work), incidence rates were calculated according to the change in glycemic status. In addition, Cox regression models were constructed for each variable of interest, obtaining crude (cHR) and adjusted (aHR) hazard ratios with their respective 95 % confidence intervals. It is important to emphasize that the selection of variables for the adjusted model was based on a careful analysis using a Directed Acyclic Graph. This revealed that only sex and age were confounding variables in the relationship between our occupational variables of interest and glycemic changes. The other potential variables were identified as mediators or non-participants in this causal relationship. Therefore, the final adjustment model included only sex as a categorical variable and age modeled using restricted cubic splines (RCS) with 3 knots to capture possible non-linear relationships.

All comparative analyses were performed using 95 % confidence intervals. Analyses were done using the ‘survival,’ ‘rms,’ and ‘msm’ packages in R version 4.1.0 or higher.

### 2.6 Ethical aspects

For the realization of this study, necessary authorization was obtained from the clinic to access and use the workers' database. Additionally, the project received approval from the Ethics Committee of the Toribio Rodriguez de Mendonza University of Amazonas, Peru.

The clinic's data were delivered anonymously, without including information that could directly identify the workers. This measure ensures the privacy and confidentiality of the participant's data, a fundamental aspect of research ethics. Furthermore, access to these data was limited exclusively to the principal investigator, which minimizes the risk of a possible privacy violation. In addition, to ensure data transparency, these are freely accessible [9].

## 3 Results

### 3.1 Baseline characteristics of participants

**Fig. 1:**
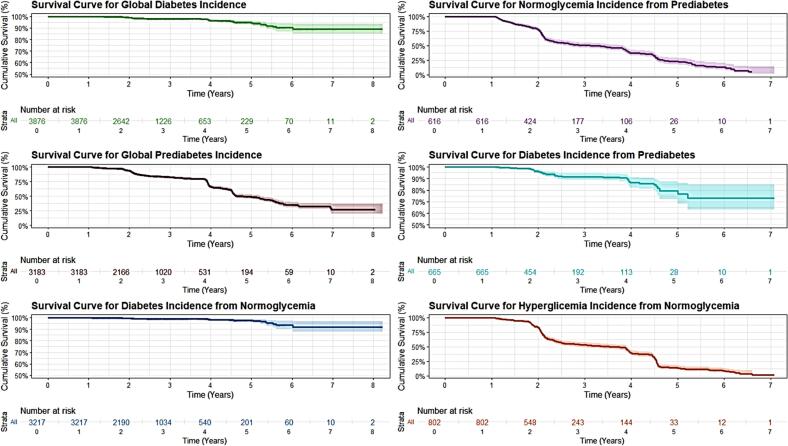
Survival curve of prediabetes and diabetes incidence and glycemic changes.

After applying the selection criteria, the study included 4200 participants, with a predominance of males (79.52 %). The mean age of participants was 39.01 years (SD 12.30). Regarding occupation, the majority worked in office settings (56.14 %), followed by physical or manual labor (35.17 %). Approximately half (52.26 %) spent up to 4 h sitting during their workday. Night work was uncommon (7.36 %), and 37 % had been in their current job for less than a year. In terms of health, 8.64 % reported a family history of diabetes, 40.17 % were smokers, and 49.62 % consumed alcohol. At the start of the study, 3.69 % of participants had a diagnosis of diabetes, and 17.07 % had prediabetes. The mean values for blood pressure, waist circumference, blood glucose, cholesterol, and triglycerides were within ranges considered normal or slightly elevated. The rest of the results can be seen in Table 1. (See Fig. 1.) (See Table 2, Table 3, Table 4.)

**Table 1:** Characteristics of participants at the baseline of the study.

**Characteristic**	***n* = 4200**
**Sex**	
Female	860 (20.48 %)
Male	3340 (79.52 %)
**Age**	39.01 (12.30)
**Occupation Type**	
Office	2358 (56.14 %)
Physical or Manual	1477 (35.17 %)
Customer Service or Sales	22 (0.52 %)
Health Professional	75 (1.79 %)
Social Services	268 (6.38 %)
**Time Spent Sitting**	
Up to 4 h	2195 (52.26 %)
More than 4 h	2005 (47.74 %)
**Night Shift Work**	
No	3891 (92.64 %)
Yes	309 (7.36 %)
**Time in the job (years)**	
Less than 1 year	1554 (37.00 %)
From 1 to 4 years and 11 months	1125 (26.79 %)
From 5 to 9 years and 11 months	561 (13.36 %)
10 years or more	960 (22.86 %)
**Family History of Diabetes**	
No	3837 (91.36 %)
Yes	363 (8.64 %)
**Smoking Status**	
No	2513 (59.83 %)
Yes	1687 (40.17 %)
**Alcohol Consumption**	
No	2116 (50.38 %)
Yes	2084 (49.62 %)
**Systolic Blood Pressure**	111.85 (12.67)
**Diastolic Blood Pressure**	72.70 (16.46)
**Waist Circumference**	91.47 (10.73)
**Blood Glucose Level**	94.81 (23.48)
**Cholesterol Level**	195.81 (37.67)
**Triglyceride Level**	144.98 (80.85)
**T2DM**	
No	4045 (96.31 %)
Yes	155 (3.69 %)
**Prediabetes**	
No	3366 (82.93 %)
Yes	693 (17.07 %)

n (%); Mean (SD)

**Table 2:** Incidence rate of glycemic changes in Peruvian workers.

Measure	Incidence per 1000 person-years (IC 95 %)	Number of Cases	Follow-up Time (years)
Incidence of T2DM	7.64 (5.97–9.32)	80	10,465.43
Incidence of Prediabetes	77.11 (71.25–82.97)	666	8637.06
Incidence of T2DM from Normoglycemia	3.89 (2.58–5.20)	34	8740.68
Incidence of T2DM from Prediabetes	26.67 (18.96–34.38)	46	1724.76
Incidence of Normoglycemia from Prediabetes	211.67 (189.14–234.20)	339	1601.55
Incidence of Hyperglycemia from Normoglycemia	80.09 (74.15–86.02)	700	8740.68

**Table 3:** Sitting time as a determining factor in glycemic changes.

Sitting time at Work	Incidence per 1000 person-years (IC 95 %)	Number of cases	Follow-up time (years)	cHR (95 % CI)	aHR (95 % CI)⁎
**Incidence of T2DM**					
Up to 4 h	2.90 (1.48–4.33)	16	5511.83	Ref.	Ref.
More than 4 h	12.92 (9.75–16.09)	64	4953.6	**4.80 (2.77–8.31)**	**2.84 (1.58–5.12)**

**Incidence to Prediabetes**					
Up to 4 h	65.65 (58.37–72.94)	312	4752.29	Ref.	Ref.
More than 4 h	91.12 (81.63–100.62)	354	3884.78	**1.50 (1.29–1.75)**	1.16 (0.98–1.36)

**Normoglycemia to T2DM**					
Up to 4 h	1.88 (0.65–3.11)	9	4782.6	Ref.	Ref.
More than 4 h	6.32 (3.84–8.79)	25	3958.07	**3.80 (1.77–8.16)**	**2.43 (1.06–5.58)**

**Prediabetes to T2DM**					
Up to 4 h	9.60 (2.49–16.71)	7	729.23	Ref.	Ref.
More than 4 h	39.18 (26.88–51.47)	39	995.53	**1.50 (1.29–1.75)**	**3.22 (1.35–7.69)**

**Prediabetes to Normoglycemia**					
Up to 4 h	276.51 (237.80–315.22)	196	708.84	Ref.	Ref.
More than 4 h	160.19 (133.93–186.44)	143	892.71	**3.83 (1.71–8.59)**	**0.68 (0.53–0.87)**

**Normoglycemia to Hyperglycemia**					
Up to 4 h	67.12 (59.78–74.46)	321	4782.6	Ref.	Ref.
More than 4 h	95.75 (86.11–105.39)	379	3958.07	**1.54 (1.33–1.79)**	**1.18 (1.01–1.39)**

⁎ model adjusted for age (RCS) and sex 95 % CI: 95 % confidence interval.

**Table 4:** Night Work as a determining factor in glycemic change.

Night Shift Work	Incidence per 1000 person-years (IC 95 %)	Number of cases	Follow-up time (years)	cHR (95 % CI)	aHR (95 % CI)⁎
**Incidence of T2DM**					
No	6.11 (4.55–7.67)	59	9657.83	Ref.	Ref.
Yes	26.00 (14.88–37.12)	21	807.6	**4.00 (2.43–6.58)**	**3.24 (1.97–5.35)**

**Incidence to Prediabetes**					
No	76.30 (70.27–82.32)	616	8073.69	Ref.	Ref.
Yes	88.75 (64.15–113.35)	50	563.37	1.12 (0.84–1.49)	1.02 (0.76–1.36)

**Normoglycemia to T2DM**					
No	3.55 (2.26–4.85)	29	8160.26	Ref.	Ref.
Yes	8.61 (1.06–16.17)	5	580.41	2.33 (0.90–6.02)	1.89 (0.73–4.91)

**Prediabetes to T2DM**					
No	20.03 (12.86–27.20)	30	1497.57	Ref.	Ref.
Yes	70.43 (35.92–104.94)	16	227.19	**3.11 (1.69–5.72)**	**3.18 (1.73–5.87)**

**Prediabetes to Normoglycemia**					
No	215.44 (191.30–239.58)	306	1420.32	Ref.	Ref.
Yes	182.09 (119.96–244.22)	33	181.23	0.72 (0.50–1.03)	**0.69 (0.47–0.99)**

**Normoglycemia to Hyperglycemia**					
No	79.04 (72.94–85.14)	645	8160.26	Ref.	Ref.
Yes	94.76 (69.72–119.80)	55	580.41	1.15 (0.87–1.51)	1.04 (0.79–1.37)

⁎ model adjusted for age (RCS) and sex 95 % CI: 95 % confidence interval.

### 3.2 Incidence of prediabetes, T2DM, and glycemic changes

The overall incidence of diabetes was 7.64 cases per 1000 person-years (95 % CI: 5.97–9.32), while that of prediabetes was considerably higher, with 77.11 cases per 1000 person-years (95 % CI: 71.25–82.97). The progression from normoglycemia to diabetes was relatively low, with 3.89 cases per 1000 person-years (95 % CI: 2.58–5.20), while the progression from prediabetes to diabetes had 26.67 cases per 1000 person-years (95 % CI: 18.96–34.38). Notably, the reversion from prediabetes to normoglycemia showed the highest rate, with 211.67 cases per 1000 person-years (95 % CI: 189.14–234.20). Lastly, the incidence of hyperglycemia from normoglycemia was 80.09 cases per 1000 person-years (95 % CI: 74.15–86.02).

### 3.3 Survival curve and glycemic changes

The graph presents six survival curves illustrating different transitions in glycemic status over time. The overall diabetes incidence curve shows a gradual decrease in survival (i.e., remaining diabetes-free) over 8 years, with a more pronounced drop after the fourth year. The overall prediabetes incidence curve shows a faster and more marked decrease, especially from the third year onwards, indicating a high rate of prediabetes development in the studied population.

The curve for the incidence of normoglycemia from prediabetes shows the fastest rate of change, with a pronounced decrease in the first 2 years, suggesting that the reversion from prediabetes to normoglycemia is more common in the first years after diagnosis. The curve for diabetes incidence from prediabetes shows a more gradual but constant decrease. In contrast, the curve for diabetes incidence from normoglycemia shows the lowest rate of change, remaining relatively stable over time. Finally, the curve for hyperglycemia incidence from normoglycemia shows a rapid and constant decrease, indicating a high rate of progression toward states of glycemic alteration.

### 3.4 Sitting time and glycemic changes

Analyzing the incidence of glycemic alterations according to sitting time at work reveals significant patterns. The incidence of diabetes was notably higher in those who spent more than 4 h sitting, with a significantly elevated adjusted risk (aHR: 2.84, 95 % CI: 1.58–5.12). The progression from normoglycemia to diabetes also showed a significantly higher adjusted risk in the group that spends more time sitting (aHR: 2.43, 95 % CI: 1.06–5.58). For the progression from prediabetes to diabetes, the adjusted risk was even higher (aHR: 3.22, 95 % CI: 1.35–7.69) in the group that spends more time sitting. Interestingly, the reversion from prediabetes to normoglycemia was less common in those who spent more time sitting (aHR: 0.68, 95 % CI: 0.53–0.87). The incidence of hyperglycemia from normoglycemia was also significantly higher in the group that spends more time sitting (aHR: 1.18, 95 % CI: 1.01–1.39).

Regarding night work, the incidence of diabetes was notably higher in night workers, with a significantly elevated adjusted risk (aHR: 3.24, 95 % CI: 1.97–5.35). For the progression from prediabetes to diabetes, the adjusted risk was even higher in night workers (aHR: 3.18, 95 % CI: 1.73–5.87). Interestingly, the reversion from prediabetes to normoglycemia was less common in night workers (aHR: 0.69, 95 % CI: 0.47–0.99). No significant differences were observed for the rest of the variables.

## 4 Discussion

### 4.1 Incidence of glycemic changes

The results of our study reveal notably high incidence rates of diabetes and prediabetes in Peruvian workers. The overall incidence of diabetes was 33.1 per 1000 person-years, which is considerably higher than that reported in Caucasian populations (11.1–16.8 per 1000 person-years) [10,11], but similar to that observed in other minority ethnic groups in the United States such as African Americans and Hispanics (21.6–21.9 per 1000 person-years) [11]. Our findings are comparable to those reported in South Asian immigrants in the United States (33.1 vs 18.9 per 1000 person-years) [12], suggesting a similar ethnic susceptibility. However, the incidence of prediabetes in our cohort (77.11 per 1000 person-years) was notably higher than that reported in other studies, including one in South Asians in India (29.5 per 1000 person-years) [13]. This could indicate a faster progression through dysglycemic states in our population, possibly influenced by specific genetic and environmental factors.

On the other hand, it is important to highlight that a high rate of regression to normoglycemia was found from patients with prediabetes, which was 39.8 per 1000 person-years (95 % CI: 28.9–53.7). This rate was higher than the rate of progression from prediabetes to diabetes, which was 36.8 per 1000 person-years (95 % CI: 26.3–50.2). These results are interesting and contrast with the findings of Gujral et al. [12], who reported a lower regression rate in South Asian immigrants in the United States. Our data are more similar to those reported by Anjana et al. [13] in a population from southern India, where a high regression rate was also observed. Several factors, including medical interventions, could explain this tendency toward regression once a patient is diagnosed with prediabetes, as it is reflected in Peru's occupational law that every patient must be treated and controlled by the company they belong to once a diagnosis is received. These findings underscore the importance of early interventions in individuals with prediabetes, as there is a significant window of opportunity to prevent or delay progression to diabetes.

### 4.2 Comparison with other studies

Our study revealed significant associations between night work and the risk of developing glycemic alterations. Night workers showed a notably elevated risk of developing diabetes (aHR: 3.24, 95 % CI: 1.97–5.35) compared to day workers. This finding is consistent with previous studies and meta-analyses that have identified shift work, especially night work, as an important risk factor for diabetes [14,15]. For example, a meta-analysis conducted by Gan et al. in 2015 found that shift workers had a 9 % higher risk of developing diabetes compared to day workers, with an even higher risk for night workers [14].

Moreover, we observed that night work was also associated with a higher risk of progression from prediabetes to T2DM. This finding suggests that night work not only increases the risk of developing diabetes but also accelerates disease progression in those who already have an elevated risk. Interestingly, the reversion from prediabetes to normoglycemia was less common in night workers, indicating that night work not only increases the risk of developing glycemic alterations but could also hinder their reversal.

These results can be explained by the effects of night work on the dysregulation of circadian rhythm, which in turn affects glucose metabolism, insulin sensitivity, and cortisol secretion [16]. The circadian rhythm plays a crucial role in regulating glucose homeostasis, and its alteration can lead to desynchronization between sleep-wake cycles and eating patterns, which in turn can negatively affect glucose metabolism. A study by Vetter et al. in 2018 found that night work was associated with a 44 % higher risk of type 2 diabetes, which is comparable to our findings [15]. This study also suggested that the risk increased with the duration of exposure to night work, supporting the idea of a cumulative effect of shift work on metabolic risk.

Furthermore, night work is often associated with irregular sleep patterns, changes in eating habits, and decreased physical activity, all contributing to metabolic risk [17]. For example, night workers often have limited access to healthy food during their shifts and may resort to processed or calorie-rich foods. They may also have difficulties maintaining a regular exercise schedule due to fatigue and irregular work hours.

On the other hand, our study revealed a significant association between prolonged sitting time at work and the risk of developing glycemic alterations. Workers who spent more than 4 h sitting per day showed a notably elevated risk of developing T2DM compared to those who spent less time sitting. This finding is consistent with previous studies that have identified prolonged sedentary behavior as an important risk factor for diabetes [18,19]. For example, a meta-analysis conducted by Biswas et al. in 2015 found that prolonged sedentary time was associated with a 112 % increase in the risk of T2DM [19].

Moreover, we observed that prolonged sitting time was also associated with a higher risk of progression from normoglycemia to diabetes and from prediabetes to T2DM. These results suggest that sedentary behavior increases the risk of developing T2DM and accelerates disease progression at different stages of the glycemic continuum. Interestingly, the reversion from prediabetes to normoglycemia was less common in those who spent more time sitting, indicating that prolonged sedentary behavior could also hinder improvement in glycemic status. These results are comparable to those found by van der Berg et al., who reported that each additional hour of sedentary time was associated with a 22 % increase in the risk of T2DM [18].

These results can be explained by the negative effects of prolonged sedentary behavior on glucose metabolism, insulin sensitivity, and endothelial function [20]. Prolonged sitting time has been associated with decreased lipoprotein lipase activity, a key enzyme in lipid and glucose metabolism [21]. Additionally, prolonged sedentary behavior can reduce muscle contraction-mediated glucose metabolism, contributing to insulin resistance and impaired glucose tolerance.

In line with these pathophysiological mechanisms and their implications for workplace health, our findings gain additional relevance when compared with recent research from other developing nations. A 2024 study by Basu et al. [22] analyzing nationwide data from India found similar patterns of diabetes risk factors, highlighting the importance of early detection and prevention strategies in workplace settings. This aligns with our observations about the need for targeted interventions, especially in occupational groups with high exposure to sedentary behavior and night work. Furthermore, Lahariya [23] demonstrated the effectiveness of integrating diabetes prevention programs into primary healthcare systems, which could serve as a model for workplace health initiatives. Recent advances in diabetes management, as reviewed by Gokalani et al. [24], suggest that workplace intervention programs could benefit from incorporating preventive strategies and support for treatment adherence, particularly in populations exposed to occupational risk factors like those identified in our study. These emerging therapeutic approaches could be especially relevant for workers with prediabetes or early-stage diabetes, where appropriate intervention could prevent or delay disease progression.

### 4.3 Contribution to the field

This study contributes significantly to understanding occupational risk factors for developing T2DM and prediabetes, focusing specifically on night work and prolonged sitting time. By highlighting the association between these factors and an increased risk of glycemic alterations, our findings enable public health and occupational health professionals to target preventive interventions more effectively. This information is crucial for designing specific prevention programs and efficient resource allocation in the workplace.

Our work provides robust evidence of the negative impact of prolonged sitting time at work on the risk of T2DM and prediabetes. These findings reinforce the growing literature on the detrimental effects of sedentary behavior and provide specific data in the occupational context. This contribution is particularly relevant in the current era, where many jobs involve long periods of sitting. Our results can drive the development of workplace policies and practices that encourage reducing sedentary time, such as the implementation of standing workstations, regular active breaks, or the promotion of physical activity during the workday.

Furthermore, our study provides crucial evidence on the metabolic effects of night work. These findings have significant implications for occupational health management in industries that require night work, such as healthcare, manufacturing, and emergency services. Our results can drive the development of mitigation strategies for night workers, such as optimized work schedules, tailored lifestyle interventions, and early detection programs for metabolic alterations. Moreover, these findings can inform labor and public health policies related to shift work and its health impacts.

Collectively, our findings on night work and prolonged sitting time as risk factors for T2DM and prediabetes have important implications for preventing and managing these conditions in the workplace. By identifying these modifiable risk factors, our study provides a solid foundation for developing comprehensive workplace interventions. These results can guide the design of more effective workplace wellness programs, including promoting physical activity, education on metabolic health, and modifying work environments to reduce sedentary behavior. Additionally, our findings underscore the need for closer collaboration between occupational health professionals, employers, and public health policymakers to effectively address the growing challenge of diabetes in the working population, especially those with unconventional work schedules or sedentary jobs.

### 4.4 Limitations of the study

This study presents several strengths, including its longitudinal design, which allows for establishing temporal relationships between occupational factors and glycemic changes, and its large sample size, which increases statistical power and precision of estimates. Additionally, including multiple occupational variables provides a comprehensive view of work-related risks associated with glycemic changes. However, the study also has limitations that should be considered. Firstly, the studied population is concentrated on workers from a specific region of Peru, which may limit the generalization of results to other populations or work contexts.

A potential limitation of this study is categorizing the sitting time variable into >4 h and ≥ 4 h. This categorization is because the information was collected in this manner during routine occupational evaluations, which prevented a more detailed or continuous analysis of this variable. However, it is important to highlight that this categorization is supported by scientific literature [20]. Therefore, although the dichotomization limits the analysis of the dose-response relationship between sitting time and diabetes risk, the cut-off point used is consistent with existing evidence.

Notably, our study period (2014–2021) encompassed the COVID-19 era, which warrants specific considerations. During 2020–2021, several data collection limitations were observed due to pandemic-related restrictions, including 1) a temporary reduction in routine occupational evaluations due to remote work arrangements and social distancing measures, 2) potential delays in scheduled periodic assessments, and 3) a possible selection bias as some workers may have postponed their evaluations due to concerns about viral transmission. Nevertheless, the longitudinal nature of our study, with extensive follow-up both before and after the pandemic's most critical period, enables us to maintain the robustness of our primary findings. Furthermore, measures implemented by companies to continue occupational evaluations (such as biosafety protocols and scheduled appointments) helped minimize the loss of follow-up. Notably, we did not observe significant changes in incidence rates before and during the pandemic, although this could be subject to more detailed analysis in future studies.

## 5 Conclusions

The present study revealed a high incidence of T2DM and prediabetes in Peruvian workers, as well as a considerable rate of regression from prediabetes to normoglycemia, underlining the dynamic nature of intermediate glycemic states. Significant associations were found between the risk of glycemic alterations and two specific occupational factors: prolonged sitting time and night work. Workers who spend more than 4 h sitting per day and those who perform night work showed a notably elevated risk of developing T2DM. These results highlight the importance of considering these aspects of the work environment as critical factors in diabetes prevention and management strategies.

Based on these findings, we recommend implementing workplace-specific T2DM prevention programs focused on reducing sedentary time and mitigating the effects of night work. These interventions should include strategies to promote physical activity during the workday and address the unique challenges of night shifts. Additionally, we suggest implementing early detection programs for glycemic alterations, especially for these high-risk groups. At the policy level, we recommend the consideration of these occupational risk factors in public and occupational health guidelines. Finally, we urge further research to evaluate the effectiveness of these interventions and to further explore the underlying mechanisms of the observed associations between these occupational factors and the risk of these diseases.

## Funding

This study was financed by Vicerectorado de Investigación de la Universidad Nacional Toribio Rodríguez de Mendoza de Amazonas.

## Informed consent

Since this is a secondary data analysis, informed consent was not required.

## CRediT authorship contribution statement

**Víctor Juan Vera-Ponce:** Writing – review & editing, Writing – original draft, Software, Resources, Methodology, Investigation, Formal analysis, Data curation, Conceptualization. **Fiorella E. Zuzunaga-Montoya:** Writing – review & editing, Writing – original draft, Project administration, Methodology, Investigation. **Nataly Mayely Sanchez-Tamay:** Writing – review & editing, Writing – original draft, Visualization, Validation, Investigation. **Juan Carlos Bustamante-Rodríguez:** Writing – review & editing, Writing – original draft, Resources, Investigation. **Carmen Inés Gutierrez De Carrillo:** Writing – review & editing, Writing – original draft, Supervision, Methodology, Funding acquisition.

## Declaration of competing interest

The authors declare no conflict of interest.

## Data Availability

The data supporting the findings of this study can be accessed through the following link: https://doi.org/10.6084/m9.figshare.27098296
